# Cooperative Vehicular Traffic Monitoring in Realistic Low Penetration Scenarios: The COLOMBO Experience

**DOI:** 10.3390/s18030822

**Published:** 2018-03-09

**Authors:** Paolo Bellavista, Federico Caselli, Antonio Corradi, Luca Foschini

**Affiliations:** DISI—Department of Computer Science and Engineering, University of Bologna, 40136 Bologna, Italy; f.caselli@unibo.it (F.C.); antonio.corradi@unibo.it (A.C.); luca.foschini@unibo.it (L.F.)

**Keywords:** vehicular traffic estimation and management, vehicular communications, low penetration rate, cooperative vehicles, iTETRIS, performance evaluation

## Abstract

The relevance of effective and efficient solutions for vehicle traffic surveillance is widely recognized in order to enable advanced strategies for traffic management, e.g., based on dynamically adaptive and decentralized traffic light management. However, most related solutions in the literature, based on the powerful enabler of cooperative vehicular communications, assume the complete penetration rate of connectivity/communication technologies (and willingness to participate in the collaborative surveillance service) over the targeted vehicle population, thus making them not applicable nowadays. The paper originally proposes an innovative solution for cooperative traffic surveillance based on vehicular communications capable of: (i) working with low penetration rates of the proposed technology and (ii) of collecting a large set of monitoring data about vehicle mobility in targeted areas of interest. The paper presents insights and lessons learnt from the design and implementation work of the proposed solution. Moreover, it reports extensive performance evaluation results collected on realistic simulation scenarios based on the usage of iTETRIS with real traces of vehicular traffic of the city of Bologna. The reported results show the capability of our proposal to consistently estimate the real vehicular traffic even with low penetration rates of our solution (only 10%).

## 1. Introduction

The Intelligent Transportation Systems (ITS) research field has attracted careful consideration of the research and industrial communities in the last years, also because of its enormous potential in terms of economic and societal impact. It is widely recognized that, through the usage of vehicular communications, many novel valuable services can be enabled, spanning from enhanced safety of road users and provisioning of advanced applications to drivers/passengers (e.g., best path determination and infotainment), to optimized vehicular traffic flow management in order to efficiently exploit the available road infrastructure. Indeed, it is well accepted that the identification of good and economically sustainable traffic management solutions is becoming one of the top priorities for future Smart Cities [1,2]. In particular, in dense urban environments, vehicular traffic congestions are a major problem that is documented to generate serious environmental and economic costs, as well as to lead to user frustration [3,4].

Given the recent advancements and the strong industrial interest in the related technologies, with ever-increasing frequency, ITS solutions for traffic optimization make extensive use of Vehicular Ad-hoc Networks (VANETs), which enable different forms of vehicular communications: between vehicles (V2V), between mobile nodes and fixed infrastructure points (V2I), and a combination of the two (V2X). To successfully apply VANET-related technologies to the field of vehicular traffic monitoring and surveillance in smart cities, however, some relevant and still open technical challenges have to be overcome, such as the low penetration rate of vehicles with wireless communication capabilities or at least of vehicles willing to trustfully serve in a traffic surveillance cooperative service, a scenario that will likely persist in the near future. In addition, even the future envisioned scenario of high concentration of communication-enabled vehicles poses serious technical challenges, e.g., because of wireless medium congestion and consequent performance degradation deriving from high quotas of vehicles locally participating to a cooperative service via their wireless connectivity capabilities. Moreover, to enable ready-to-use and reliable traffic management optimizations, e.g., via advanced traffic light control algorithms that alleviate traffic congestion based on traffic monitoring data and predictions, there is the need of traffic surveillance solutions that are at the same time: (i) easily and cost-effectively deployable and (ii) precise and robust enough to solidly support delicate traffic management tasks.

Several interesting solutions for traffic surveillance solutions are available in the recent related literature, but tend to work under the strongly simplifying assumption of complete penetration rate of communication capabilities in the considered vehicle population [5] or, when considering partial penetration rates, they assume that the rate is statically known and that communication-enabled vehicles are uniformly distributed over the road network [6]. These assumptions are far from reality in any case of practical industrial applicability. In addition, the existing traffic surveillance protocols generally collect only a limited range of monitoring data (typically only the density of vehicles over a target area or the number of vehicles travelling on a specific road, sometimes without the capability of distinguishing the fractions moving in the different directions). Moreover, the quality of the collected monitoring data strongly depend on the penetration rate of the proposed solution (both communication capabilities and adoption of the proposed protocol by targeted participants), thus being of limited value as traffic flow indicators to enable traffic management strategies in realistic smart city scenarios.

Given the above limitations, we have worked to develop an original solution for traffic surveillance, by taking into special account low penetration rate of our proposal (considering both the practical aspects of limited fraction of vehicles with wireless connectivity and limited fraction of communication-enabled vehicles willing to participate to our collaborative surveillance service) and the goal of collecting a large array of different monitoring data about vehicles in targeted areas of particular interest. In particular, our proposal exploits V2I communications between vehicles and Road Side Units (RSUs), deployed at selected traffic lights at the occurrence of relevant intersections to collect various information about local traffic status. To enable a precise and robust view of traffic flow performance and indicators, necessary to enable effective and dynamic traffic management, our solution also discriminates the different roads converging to the intersection and in which direction the vehicles are travelling on these roads. The solution originally presented here is also a follow-up of and exploits the research experience made thanks to the research activities accomplished within the framework of the EU COLOMBO project [7], which aimed at optimizing road network usage and at alleviating traffic congestions through VANET-based optimization of decentralized traffic light control and management.

To validate and assess the performance results achievable by our proposed solution, the paper also contributes to the literature in the field with a thorough reporting, analysis, and comparison of several performance indicators of our original technique with regards to the existing literature: the reported performance results have been collected over realistic simulation scenarios deriving from the usage of the state-of-the-art iTETRIS [8] framework, fed with real traces of vehicular traffic measured in the city of Bologna (a medium-size urban environments of around 450,000 inhabitants). Our contribution to the community in the field also includes the availability of the code for the proposed solution and of our original extension for improved usability of the iTETRIS framework, as described in the following sections; the interested readers may refer to http://lia.disi.unibo.it/ Research/iTetrisUniboExt/ for additional details and downloads.

## 2. Cooperative V2X Traffic Surveillance: Background and Related Work

The currently available and traditional traffic surveillance solutions usually exploit infrastructure-based detection devices such as inductive loops, pressure sensors, and traffic surveillance cameras, to be deployed on monitored roads [9]. These solutions, moreover, generally make use of a centralized architecture with a single point (typically hosted in the cloud) that gathers the global state of the vehicular traffic for the whole targeted area and uses it to make centralized predictions and decisions, then to be enforced in a distributed way via control commands to the different covered sub-areas. That has a non-negligible cost in terms of wired/wireless communication, often based on the use of cellular connectivity with expected global coverage, which leads to significant deployment and management costs [10,11].

In parallel to the aforementioned traditional traffic surveillance solutions, we are aware of other services based on the collection of location information from connected smartphones, ad mobile apps running atop, that have expanded dramatically in the last years. Some of the most well-known examples are Google Traffic and Waze. These systems are currently primarily focused on collecting gross traffic density estimations at a certain road trunk. Those estimations are traditionally made available to registered (public) users providing the data; only recently, these players (e.g., Google) are making their information available also to city administrators and that could pave the way to their use for traffic management purposes. In this paper, we focus on vehicular communications that have the different goal of providing a precise, finer-grained, and punctual estimation of the traffic (e.g., number of cars and velocity variability) at the intersection to be used to enable advanced strategies for traffic management, e.g., for optimized traffic light management as in the COLOMBO project.

More recent solutions targeted at collecting information for traffic management systems are shifting the focus on local data collection through the use of local traffic control protocols. This has the potential to eliminate the need for a centralized collection and processing point: the goals are to reduce complexity and costs associated with traffic surveillance, as well as to increase reliability and scalability of the adopted solutions, while retaining appropriate levels of quality/performance indicators, such as accuracy and precision. These less traditional proposals are exploring the possibility to actively involve the participating vehicles, their growing communication capabilities, and their on-board sensors, enabled by the significant advancements in vehicular networking (and its decreasing costs) and related standardization efforts, such as European Cooperative ITS (C-ITS) or American Dedicated Short Range Communications (DSRC) [12]. Furthermore, if compared with traditional sensors, which are prone to inaccuracy issues, require periodic maintenance, and have to be placed on every road that needs to be monitored, in principle the vision of connected vehicle collaboration can offer better accuracy and can be used to collect information in the entire road network at very limited costs.

The cooperative traffic surveillance solutions currently available in the literature can be classified according to several parameters: their architecture may be centralized, when all collected data from participating vehicles are sent to a central elaboration point, or decentralized, when monitoring data are distributed among the nodes; the protocols can use direct communication between vehicles, or can rely on groups of nodes with similar characteristics/features; the collected information may be either used locally or shared globally within the whole monitored area; finally, the type of collected data may be used as a classification criterion, from the simple visibility of the concise indicator of vehicle density in the targeted area, to more detailed monitoring information, e.g., including position, speed, acceleration, break usage, capable of discriminating the different roads and vehicle directions.

As stated above, traffic surveillance systems can collect different types of information about the flow of vehicles depending on the particular type of sensors used, such as number of vehicles, their density inside a specific area, or the average speed on a specific street. Classic induction loops and pressure sensors installed on the road surface provide only a limited subset of the potential information of interest, for example, to optimize traffic light management. The use of wireless transmission interfaces that can share the large amount of data collected by the sensors already installed inside the single vehicles can enable the collection of more detailed information, which can be used to develop new and more accurate traffic control algorithms. However, up to now, this type of sensors has suffered from the significant drawback of low penetration rate in the current vehicle population. Although this problem should gradually become less relevant, it is nevertheless important to focus on the collection of traffic surveillance information that has a low dependence on the number of vehicles actively cooperating to their collection. Indeed, let us note that (as it will be shown also by our reported experimental results in this paper) the precision and accuracy of some types of collected data, such as the number of vehicles, are more sensitive on the penetration rate, while others, like the average speed, are less conditioned; also this element could represent a significant design/selection choice when considering cooperative traffic surveillance systems.

### Related Work

Even if VANET is a very active research area, the majority of the attention so far has been focused on new and optimized routing protocols to overcome the numerous challenges posed by these mobile networks. In this paper, we focus only on traffic surveillance solutions, an area in which most literature has concentrated on estimating very basic indicators such as the number of mobile vehicles inside a targeted area, usually called density, thus gathering only a general concise overview of the traffic state. The methods used to collect the information can make use of a clustering approach to group the nodes together if they are moving in the same general direction [13] or if they are close to a fixed point [14]; or it can make use of direct communications between the participating nodes and, when present, the infrastructure support points (e.g., RSUs), such as in [15,16,17]. While the goal of the present paper is the presentation of a quantitative comparison and benchmark of notable existing proposals, for a qualitative and more exhaustive review of existing efforts in the field, we refer interested users to the survey [18].

Among the traffic surveillance solutions available in the literature, we have decided to consider three relevant protocols that have become relatively well-known in the community in the field and that are sufficiently well described in the corresponding papers to allow us to implement them in our simulation work (reproducibility of their performance results). In addition, as it will be clarified in the following sections, these three protocols are fairly comparable with our original proposal because they share some similar characteristics and features.

The first one is the MobSampling [16] protocol. MobSampling has been designed to estimate vehicle density over a targeted area: a source node (either fixed or mobile) sends a message to the targeted area to start the sampling process; the first vehicle receiving this message becomes the sampler and has the task of periodically estimating the density in the area. The protocol also considers the possibility that the sampler node leaves the targeted area, by including a role switching procedure to transfer the sampler role. The density estimation process in MobSampling works as follows: the sampler randomly selects when to trigger the sampling, with a uniform distribution with its average value equal to T seconds; in each sampling period, the sampler broadcasts a POLL message that includes the location of the center and the radius of the targeted area; if a vehicle inside the targeted area receives the POLL message, it will answer with a REPLY message after a random delay (to avoid broadcast storms towards the receiver); finally, the sampler node computes the instantaneous density as the ratio between the number of received REPLAYs to the last POLL message and the road length in the targeted area.

The next two selected protocols, discussed in [17], were initially developed for ecology-oriented applications to estimate wild animal population and then adapted to traffic surveillance applications. Both produce an estimation of the number of vehicles inside the targeted area, by assuming that every vehicle periodically sends status messages, such as the safety messages used in DSRC.

In particular, the Capture-Recapture protocol estimates the total population by considering only a small number (at least two) of independent samples of “captured” vehicles. Considering the method with only two samplings, as in [17] and in this paper, the estimation of the population *N* is obtained with the formula *N = mn/r*, where *m* is the number of distinguished nodes counted in the first sampling, *n* is the count of the second sampling, and *r* is the number of vehicles counted in both samplings. In vehicle-oriented traffic surveillance, a vehicle is counted if one of its messages is successfully received by a neighbor vehicle. To obtain appropriate and valid results with such a protocol, two assumptions are typically adopted: (i) a *closure assumption* dictates that the population should remain stable while the estimation samplings are performed; and (ii) a *homogeneity assumption* requires that the probability of being counted is equal for every node in the observed population. In the VANET scenario, the first assumption can be considered satisfied if the time between two samplings is small enough, while the second assumption is not accurately met because the message reception probability is significantly affected by the distance between sender and receiver, by their position, and by the road topology, among the others.

Given the above limitations, to obtain a valid estimation even when there are variations in the capture probability, [17], studies the performance of another protocol, characterized by a non-parametric estimation model designed to work under various capture probability distributions, which is based on the Jackknife estimators. In particular, the estimation of the population size is obtained through the collection of the identifiers of the captured vehicles (for example, their MAC addresses) in independent sets over multiple independent sampling. After *t* collection rounds the protocol computes the total number of different sampled nodes, i.e., *S*. This parameter is biased, with its bias decreasing with the increase of *t*. To remove the bias of *S*, a series of estimators are used, which take into account the number of times the vehicles are captured in *t* successive rounds. Different approximations are possible, depending on the specific estimator used, called *N*1 to *N*5, which have increasingly less bias but higher mean square error. Additional details about the associated algorithm can be found in [17,19].

## 3. Our Original Traffic Surveillance Solution for Low Penetration Rate Scenarios

As a follow-up of the relevant research results achieved within the framework of the EU COLOMBO project, we have worked to design, implement, and validate original traffic surveillance protocols with the aim of collecting a large set of monitoring data about traffic status near to intersections. The final goal is the identification (and comparative quantitative evaluation) of decentralized algorithms and protocols for cooperative traffic surveillance that are able to generate reasonably accurate values of a series of indicators, useful for effective traffic light control algorithms.

According to these general guidelines and by considering, since the beginning, realistic and practical usage scenarios for actual traffic optimization, we have designed an original local protocol called Traffic Surveillance for Low Penetration Rate (TraSLowPeR): the TraSLowPeR collection areas are limited to the transmission range of each RSU node, located at relevant intersections; in fact, in our preliminary work, we established that the limited traffic monitoring data in the RSU coverage area are sufficient to obtain meaningful indicators about traffic status near intersections, while the additional information obtained in the expanded coverage area provided by multi-hop routing protocols were not necessary to the aim of traffic surveillance for our local traffic light optimization system [20].

In our targeted use cases only few communication-enabled vehicles are typically inside their respective transmission range, so we deemed appropriate the definition of a direct communication protocol between RSUs and all the vehicles inside their transmission range, with the goal of creating a very detailed view of the traffic flows at the central point of the RSU for each intersection locality. Given the focus on locality of our TraSLowPeR solution, we discarded the use of collaboration between vehicles for the collection of the traffic information (examined in previous papers such as [20,21], since we believed redundant the use of clustering techniques to group the nodes together for the traffic surveillance and traffic light management application domains. We also observed that, in our typical use cases, the average cluster dimension would be close to one, since the size is directly proportional to the penetration rate (low in our challenging assumptions), making the exploitation of clustering techniques unessential. Furthermore, we believe counter-productive the use of data aggregation mechanisms among mobile nodes because of the limited quantity of data available that TraSLowPeR requires to transmit (see below); instead, we preferred the transmission of every information, locally available on board of each vehicle, to the RSU, which can be further refined with data fusion practices to create high-level indicators inside the RSU, if needed.

However, since our direct transmission mode can require large bandwidth if used in high-penetration rate scenarios, we have carefully reduced any communication management overhead by adopting a simple coordination technique to avoid transmission surges that can briefly saturate the channel, leading to high collision rates. This is achieved through two different mechanisms. On the one hand, we limit the transmission opportunities of vehicles by restricting them to send messages only as a reply to an appropriate request received from a RSU (otherwise, they are always kept in an idle status). On the other hand, we separate in time and space the communications of mobile nodes traveling on the different roads leading to the considered intersection, thus reducing the sources that can simultaneously transmit. This separation is obtained by specifying an exact targeted road and direction in each request message sent by the RSU, which will be taken into consideration only by the appropriate nodes.

In finer details, each RSU updates its traffic status indicators by periodically broadcasting request messages and exploring every road leading to its controlled intersection in a round-robin way. A request message sent by the RSU contains two data items: (i) the road that is currently being polled and in what direction (approaching or leaving the intersection), (ii) the time allotted by the RSU for the replies from the activated nodes. To identify a road, RSUs and nodes can potentially use many different conventions: from the cardinal direction of the road with regards to the intersection, to the actual name of the road, from pre-defined raw unique identifiers to increasing counters starting from the north direction and clockwise, etc. In our prototype and in the following experiments, for the sake of clarity and simplicity, we used road-name-based identifiers.

When a request is received, the receiving node checks if it is traveling on the “currently investigated” road: if so, it will reply to the message on a random instant inside the time windows specified by the message to reduce the chance of transmission overlapping with other nodes; otherwise, it will discard the message. The reply sent by a node contains the node identifier, its current position, speed, and acceleration, and its mean speed and acceleration over a selectable (configurable) interval.

Each request message defines a reply window within which targeted vehicles have to send their updated status to further minimize the chance of interference among nodes that travel on different roads: this time allotted for the replies is dynamically adapted according to the specific road network and/or to the current traffic status, e.g., by allowing more time for the streets with multiple lanes and in case of heavy traffic.

**Figure 1:**
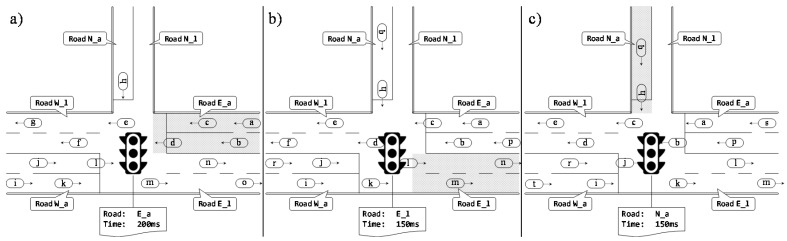
TraSLowPeR sample operation on a three way intersection. In (**a**) the RSU sends a request message limited to the road east arriving (E_a), with a reply windows of 200 ms. The nodes a, b, c, and d will then reply to the message. After the reply window elapses, the RSU will send a new request message, (**b**), for the road east leaving (E l). Next the RSU will probe the road north arriving, (**c**). The TraSLowPeR RSU will continue to sample all the roads converging in its intersection in a round robin fashion.

To practically show the primary idea behind our TraSLowPeR protocol, Figure 1 depicts a sample execution of it over a simple road topology and for a single intersection. The most relevant information extracted by the RSU from the messages exchanged through our protocol is the number of vehicles and their average speed for every road in the considered intersection and the aggregated values for the entire area controlled. TraSLowPeR is also able to distinguish the flows of vehicles in every street as divided between the traffic leading to and departing from the intersection. The total number of vehicles inside the coverage area of the RSU is obtained by considering any vehicle replaying to a request message on the current round of requests. These indicators have demonstrated to be sufficient and the most relevant ones for the specific purpose we had, i.e., optimization of traffic lights to reduce traffic congestion, but we have also experimented employing all the monitoring data collected by TraSLowPeR to extract additional higher-level performance metrics, such as estimation of queue length or number of stops required by a vehicle to pass the surveilled intersection [21].

Let us conclude this section by noting that in the reminder of the paper we will deliberately not consider security and privacy issues because the focus of the present work is on the performance evaluation of traffic surveillance solutions while considering communication aspects only and adopting a simulation-based approach. At the same time, by recognizing the importance of that aspect, we briefly note that the TraSLowPeR protocol itself is completely local and requires maintaining at the RSU only a soft state (stored for a very limited time span) about wireless network identity of the cars in the surroundings. As for other solutions and protocols considered in our performance comparison, instead, all of them completely neglect security/privacy, by considering related aspects as to be targeted in a second step.

## 4. TraSLowPeR Implementation Experience over iTETRIS

We have implemented our protocol to work over the iTETRIS framework, a platform specifically designed to simulate Intelligent Transportation Systems (ITS) scenarios by integrating, through a central component used for synchronization and coordination, two widespread and community-recognized simulators: the ns-3 communication network simulator [22] and the SUMO road traffic simulator [23]. The iTETRIS framework supports the development of user applications that can concurrently interact with both simulators, thus allowing a better evaluation of the implemented protocols thanks to the real-time feedback possible between network and road traffic simulations, at each simulation step, as described below.

While developing our solution, we have also extended the general-purpose iTETRIS simulation framework, by adding an interface that can be used as the foundation for new user applications to facilitate the implementation of new protocols while avoiding most of the complexity of iTETRIS. In fact, notwithstanding its potential, iTETRIS is recognized to be scarcely usable by the community in the field, as also shown by its slow adoption. The main goal of the developed interface is the separation of all the iTETRIS-specific interactions from our protocol specification, thus also improving possible portability over different simulation platforms. Our original iTETRIS extension is available for download at http://lia.disi.unibo.it/Research/iTetrisUniboExt/, together with some additional implementation details, which are not central to the scope of this paper.

In particular, our user application is organized into two layers: (i) a common layer shared by all the different protocols that manages the interactions with the framework and offers an API to send and receive messages; and (ii) a protocol layer that defines the logic of the specific protocol interacting with the API of the common layer. In iTETRIS, unlike in classic network simulators, the simulation is time-discrete, to allow the interaction between the various sub-simulator parts. At every time step, the central coordination component of iTETRIS sequentially commands the execution of the two sub-simulators and of the iTETRIS-based applications on top. This behavior exhibits significant advantages in terms of modularity and extensibility of the sub-simulator and the integrated framework, but introduces delays in the interaction between vehicles and RSUs, which can lead to degraded performance if compared with each of the sub-simulator separately.

To compare the performance of TraSLowPeR with the three protocols mentioned in Section 2 while running over similar conditions, we have originally implemented also MobSampling [17,19] in iTETRIS. While this implementation has maintained the protocols’ logic exactly as presented in the related papers (as well as the same configuration parameters), the very few minor variations listed below were introduced to have a fair comparison with our solution. By focusing on our MobSampling protocol implementation in iTETRIS, we assigned the sampler role to the RSU and centered the target area to its position: this eliminates the need of a starter node and of a role switching procedure. In addition, we selected to log the number of vehicles reached by the protocol and, to better compare it with the results obtained from the other solutions, we chose to use the instantaneous value (not the average value as in the original proposal). The iTETRIS implementation of the other two protocols did not require specific adaptations: we only assigned the estimation task to the RSU node, which will periodically approximate the number of vehicles inside its transmission range. Finally, since one of the protocols has the goal of determining vehicle density over a targeted area, while the other two monitor the number of vehicles, in the quantitative comparison that follows, we have reported for all cases the vehicle number indicator to homogenize the achieved results; note that the density is computed as the ratio between the number of vehicles and the length of the corresponding road segment where the number has been measured.

## 5. TraSLowPeR: Experimental Performance Comparison against Other Traffic Surveillance Protocols

A central and strongly original aspect of our TraSLowPeR-related research is the willingness to analyze and evaluate the performance of all considered protocols under realistic conditions, by using real traffic flow data obtained from the actual sampling of real intersections inside a road network based on real intersections in the city of Bologna. Two different scenarios where investigated, named Pasubio and Andrea Costa (from the actual names of the primary roads included), which represent two cross intersections of multiple lane roads regulated by traffic lights, particularly interesting in the city of Bologna because characterized by high traffic during the rush hours, with frequent slowdowns and tailbacks.

Since both the iTETRIS framework and the examined traffic surveillance protocols rely on pseudorandom number generators, we executed 20 different simulation runs with the same configuration parameters, altering each time the configuration of the employed random number generator (used at the iTetris level to generate the random instants for sending reply messages from the node to the RSU and at the ns-3 level to evaluate if messages collided), and we averaged them to obtain the final results. About protocol configurations, we have selected the parameter values shown as optimal in the original papers (see Table 1) for all simulations. In fact, digging into the papers presenting the three protocols we compared to, they neither detail the processes to optimally tune these parameters nor they report about any guarantee on the convergence of such tuning process to an optimal value. Consequently, also for this comparison motivation, we decided to consider the impact of the values of these parameters on performance outside the scope of this paper.

In particular, the configuration settings used for the iTETRIS simulator were 50 ms as time step, ns-3 version 20 configured to use the IEEE 802.11p protocol at 5.9 GHz with 6 Mbps bandwidth, with a transmission power for the RSU and the vehicles of 20 dBm, which gives a transmission range of approximatively 170 m. The Jackknife estimator protocol has different outputs depending on the type of estimator used; for the sake of briefness, we present here only the results obtained with the *N*1 estimator, which demonstrated to be the best one in the considered scenarios in our preliminary experiments; anyway little performance difference was observed for different estimators in all the tested scenarios.

To evaluate the performance of all the four traffic surveillance solutions, we used 30 min of real traffic traces collected during the morning rush hours period that provides a maximum vehicle density of about 100÷120 nodes inside the communication range of the RSU. To improve the readability of the reported charts, also by removing border effects that are not significant for the regular and continuous working of the protocols over real application scenarios, we present only the values collected between 500 s and 1500 s. Different penetration rates are simulated by randomly assigning wireless connectivity capabilities only to the selected percentage of vehicles (all connected vehicles collaboratively participate to the protocols), so that all the nodes (either connectivity-equipped or not) are still inside the simulation and “moved” by the mobility sub-simulator SUMO. We considered penetration rates of 100, 50, 20, and 10%.

**Figure 2:**
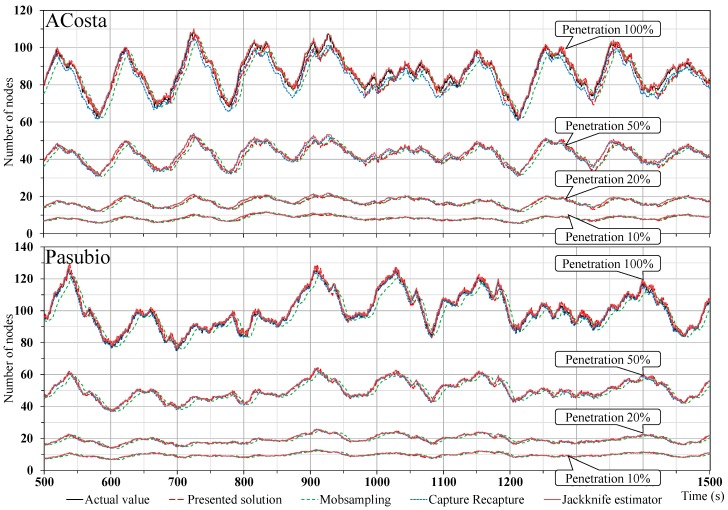
Total number of nodes determined over time by the protocols for different penetration rates in the two tested scenarios.

Figure 2 shows the traffic indicator “number of vehicles” determined by the different protocols at the variation of the penetration rate. The results obtained by the different solutions are very close, with little distance from the real number of vehicles in any case. This uniformity in the output of these different protocols depends on the relatively low vehicle density inside the target areas, which settles itself at about 80÷90 nodes. For example, the performance of the capture-recapture and the Jackknife estimator protocols is similar when the vehicle density is not such to fill the wireless medium with the status messages, which is also verified in [17]. As expected, the number of nodes is directly proportional with the penetration rate: this correlation can be clearly seen in the figure, where the indicator values determined for the 50% rate are approximately half of the ideal number of present vehicles (the same applies to the other cases).

**Figure 3:**
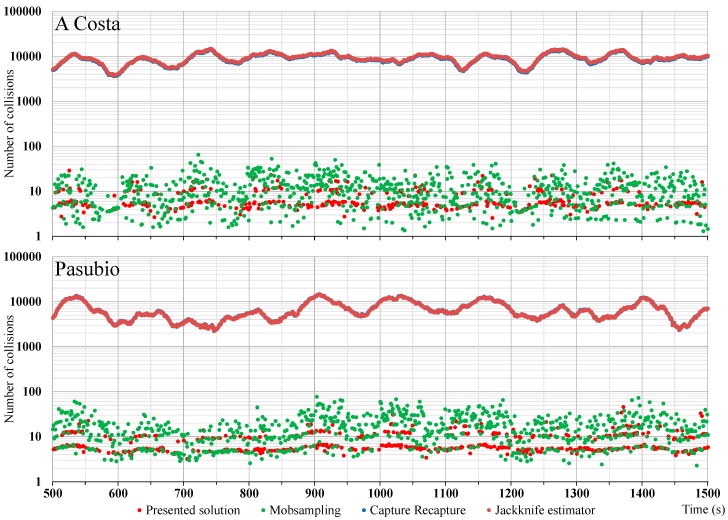
Total number of collisions over time in the most challenging case of 100% penetration rate.

Figure 3 shows the experienced message collisions suffered by the nodes for the different protocols and in the complete (100%) penetration scenario, which is the worst case for congestion, of course. The capture-recapture and the Jackknife estimator protocols, which rely on periodic status messages sent by the nodes at random instants with a frequency of 10 Hz, show a substantially higher collision rate if compared with the other solutions. While this can represent a significant problem in high node density areas, these messages are usually used by other ITS services, such as for active safety applications; therefore, when these traffic surveillance solutions are used in conjunction with other applications, they can use the existing communications to collect traffic data without introducing a high amount of new transmission overhead. The other two solutions have a comparable behavior in terms of message collisions, which is constantly lower than the other protocols. This also indicates the effectiveness of our original channel control policies to minimize the overhead of TraSLowPeR. In any case, if the RSU detects an interference with any critical applications, such as the active safety ones mentioned above, by acting as a local centralized controller it can lower the frequency used in inspecting the intersection, so to target to achieve the desired probability of collision.

**Figure 4:**
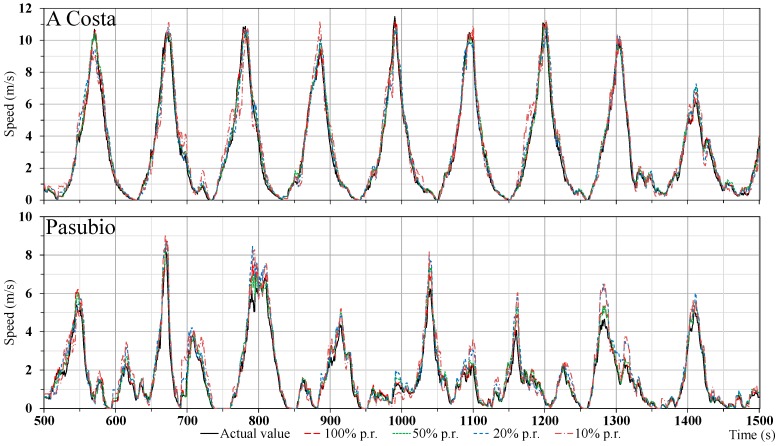
Average speed over time for different penetration rates; regardless of the considered scenario and penetration rate, the value determined by TraSLowPeR is very similar with the actual value.

As confirmed in Figure 2, regardless of the protocol used, the number of nodes has demonstrated to be unsuitable to represent a meaningful estimation of the traffic status in a realistic scenario where the penetration of the connected vehicles is typically unknown. From these evidences, we concluded that it was desirable to focus our attention on different traffic indicators, to find which estimations are less adversely affected by the variation of scenario characteristics, in particular of the penetration rate in the considered scenario. In fact, the average speed of the vehicles has demonstrated to offer a more significant, valuable, and stable representation of the monitored traffic status, as depicted in Figure 4, since it has a low dependence on the used penetration rate. In particular, the figure reports the average speed of nodes traveling on two roads leading to the intersection: it is easy to notice the periodic succession of the red and green phases of the traffic light, which are characterized by decreasing and increasing values, respectively. While the average speed computed over every vehicle in the target area is not a very significant estimation, this value cumulatively computed for each single road converging to the intersection can instead be used as a valuable and useful indication of the volume of the traffic flow.

**Figure 5:**
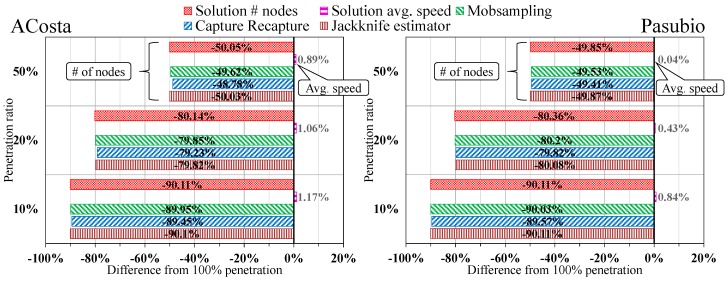
Concise overview of the performance difference in the two considered scenarios and against the complete penetration rate case.

To better summarize the different behaviors obtained by the four compared protocols, we have also defined and determined an overall indicator, shown in Figure 5, which represents the average performance difference in percentage between the reference full penetration rate and other possible (and more realistic) rates. For TraSLowPeR we report the values obtained for both the number of nodes and the average speed. The reported indicator clearly shows the linear reduction of the number of vehicles estimated, which in every protocol almost perfectly matches the selected penetration rate. Instead, the average speed of the nodes is barely affected by these variations, with a difference of less than 3% from the ground truth in the challenging case of penetration rate equal to only 10%.

## 6. Lessons Learnt and Conclusive Remarks

Our original research work in the area of traffic surveillance solutions has demonstrated how it is possible to effectively and efficiently achieve good estimations of traffic indicators that are useful for traffic light management and optimization also with simple solutions that exploit only V2I communications in the proximity of intersections. In particular, our TraSLowPeR solution has shown to outperform the existing protocols in the literature, in particular with significant advantages in terms of collision reduction, with no degradation in the estimated traffic indicators; most relevant, this is achieved even with penetration rates of our technology (vehicles equipped with wireless connectivity and willing to cooperate in the execution of our protocol) that are around only 10%. In addition, the research work has confirmed with quantitative results that the adoption of traffic indicators such as the average node speed is far more stable and almost independent of the penetration rate that can be expected in the targeted deployment environment; other indicators, such as vehicle number and density are anyway usable when it is possible to predict the penetration rate in the addressed urban areas.

The encouraging results obtained so far with the TraSLowPeR experimentation are stimulating our further research work in the field. In particular, on the one hand, we are currently extending the iTETRIS platform to reduce its resource consumption and to accelerate its simulations via more efficient (and less portable) sharing of simulation data structures between ns-3 and SUMO. On the other hand, we are evaluating other concise traffic indicators to dynamically adapt traffic light management, such as average acceleration and queuing dynamics in the proximity of intersections.

## Figures and Tables

**Table 1:** Configuration parameters of the 4 compared protocols.

Protocol	Configuration
Mobsampling	Mean poll time T: 5 s
	Transmission jitter: 200 ms
Capture recapture	Message frequency: 10 Hz
Jackknife estimator	Message frequency: 10 Hz
	Collection rounds t: 10
TraSLowPeR	Reply windows: 100÷300 ms
	Total round robin time: ACosta 1.2 s; Pasubio 1.3 s
